# The Role of Gluten in Gastrointestinal Disorders: A Review

**DOI:** 10.3390/nu15071615

**Published:** 2023-03-27

**Authors:** Sabrina Cenni, Veronica Sesenna, Giulia Boiardi, Marianna Casertano, Giuseppina Russo, Alfonso Reginelli, Susanna Esposito, Caterina Strisciuglio

**Affiliations:** 1 Department of Experimental Medicine, University of Campania “Luigi Vanvitelli”, 80138 Naples, Italy; 2 Department of Medicine and Surgery, Pediatric Clinic, University of Parma, 43126 Parma, Italy; 3 Department of Woman, Child and General and Specialist Surgery, University of Campania “Luigi Vanvitelli”, 80138 Naples, Italy; 4 Radiology Unit, Department of Precision Medicine, University of Campania “L. Vanvitelli”, 80138 Napoli, Italy

**Keywords:** gluten, inflammatory bowel disease, functional gastrointestinal disorders, celiac disease

## Abstract

Gluten is only partially digested by intestinal enzymes and can generate peptides that can alter intestinal permeability, facilitating bacterial translocation, thus affecting the immune system. Few studies addressed the role of diet with gluten in the development of intestinal inflammation and in other gastrointestinal disorders. The aim of this narrative review was to analyse the role of gluten in several gastrointestinal diseases so as to give a useful overview of its effectiveness in the prevention and management of these disorders.

## 1. Introduction

Gluten is a protein mass made of a complex network of gliadins and glutenins, which are proteins rich in glutamines and prolines found in most grains, such as barley, wheat, and rye [1,2]. Due to its high-water binding capacity and its consequent malleability and elasticity, gluten induces the formation of viscoelastic membranes, thus determining the proper consistency of dough, which allows it to be processed in bread and other foods [3,4,5]. The high content of glutamines and prolines in gliadins make them difficult to cleave, making them able to escape degradation from gastric, pancreatic, and intestinal proteolytic enzymes [3,4]. Therefore, gluten is what remains after the removal of starch, water-soluble proteins, and albumins [1].

In Western countries, the gluten dietary intake is approximately 5 to 20 g per day [3,4]. In the last decades, the literature reports an increased number of reactions following a widespread exposure to gluten [6]. Gluten-related diseases affect up to 10% of the general population and can be classified as three different disorders: IgE-mediated wheat allergy, Celiac disease (CD), and non-celiac gluten sensitivity (NCGS) [2,6]. However, there is increasing evidence that gluten can trigger an innate and adaptative immune response responsible for intestinal inflammation [7].

Notably, along with other dietary elements, gluten may contribute to the development of inflammatory intestinal disorders, such as inflammatory bowel disease (IBD), as well as functional gastrointestinal disorders (FGIDs) and concur in symptom exacerbation, although its exact role is still under investigation.

## 2. Materials and Methods

We performed a review of the literature using the PubMed database in order to find articles regarding the role of gluten acting both as a risk factor and as a therapy when it is excluded from diet in different gastro-intestinal diseases. We carried out different research regarding the association between gluten and gluten-related diseases, gluten and inflammatory bowel diseases, and gluten and functional gastrointestinal diseases. We searched the articles using key-terms commonly related to gluten-related diseases (CD non-celiac gluten sensitivity, wheat allergy, etc.), IBD (Crohn’s disease, ulcerative colitis, etc.), FGIDs (irritable bowel syndrome, functional dyspepsia, functional constipation, functional abdominal pain, Rome IV criteria, etc.) combined with terms to describe gluten and gluten-free diet (gluten, inflammation, gliadins, gluten-free diet, wheat, etc.). We reviewed a variety of different types of studies, including double or single-blind randomized placebo-controlled trials, systematic reviews, interventional, and observational studies.

## 3. Gluten and Inflammation

Inflammation is the natural response of the innate immune system to external stimuli, such as microbial pathogens and injuries [8]. When the trigger persists and the immune cells are constantly activated, the inflammatory response may become chronic and self-sustainable [8]. The aetiology of inflammation is clear and easily detectable in some health conditions, while in others it can be difficult to identify [8]. The pathogenesis of inflammation is multifactorial. Nevertheless, genetic vulnerability, psychological stress, environmental factors, and some dietary patterns have been described as potentially implicated in the development of inflammatory phenotypes [8]. There are at least 50 different types of gliadin epitopes that can have an immunomodulatory and cytotoxic role or that can impact the gut permeating activities [8]; in fact, some of these can stimulate a pro-inflammatory innate immune response and others can activate specific T cells [8].

Gliadins immune cells’ activation is not only observed in celiac patients, as described by Lammers et al. [9,10]. Indeed, their study concluded that gliadin induced an inflammatory response and, in particular, an important production of pro-inflammatory cytokines (IL-6, IL-13, and interferon-gamma) both in Celiac patients and in healthy controls, even if proinflammatory cytokine levels were higher in Celiac patients [9,10]. Similarly, Harris et al. showed that incubated peripheral blood mononuclear cells (PMBC) obtained from healthy HLA-DQ2 positive individuals produced proinflammatory cytokines, such as IL-23, IL-1beta, and TNF-α, when exposed to gliadin peptides [8,11]. These cytokines’ production was significantly higher in Celiac patients compared to healthy controls [8,11].

Accordingly, Cinova et al., in their case-control study, demonstrated that gliadin could stimulate a substantial TNF-α and IL-8 production by monocytes, principally in celiac patients, but also, to a lesser extent, in healthy control individuals [12]. Gliadin also has an important role in modifying intestinal permeability through the reorganization of actin filaments and the modified expression of junctional complex proteins [8,13]. As demonstrated by Drago et al. and Lammers et al., gliadin’s binding to the chemokine receptor CXCR3 determines a release of zonulin, an active protein, which compromises the integrity of the intestinal barrier through the rearrangements of actin filaments, ultimately leading to an altered intestinal permeability both in Celiac and non-Celiac patients [9,10,14]. In conclusion, Ziegler et al. and Junker et al. reported that amylase trypsin inhibitors, found in gluten-containing cereals, have the capacity to activate toll-like receptors, thus stimulating the release of inflammatory cytokines and inducing a T-cell immune response in both celiac and non-celiac patients [15,16].

## 4. Gluten and Gluten-Related Diseases

Celiac disease is a chronic immune-mediated enteropathy consequent to gluten intake in genetically predisposed HLA-DQ2 and HLA-DQ8 positive individuals [1]. It affects about 1% of the Western population [2,3,4,6]. The assumption of gluten activates a T-cell mediated immune response against tissue transglutaminase, which leads to an inflammation of the intestinal mucosa, resulting in small intestine villous atrophy, increased intestinal permeability, and consequent malabsorption of micro and macronutrients [3,4,6,8,17]. Many patients complain about gastro-intestinal symptoms, such as diarrhea or steatorrhea, abdominal pain, loss of weight, and failure to thrive, especially in the paediatric population [1]. Nevertheless, CD can also manifest itself with other conditions, such as anemia, infertility, reduction in bone mineral density, resulting in osteoporosis, and neuro-psychological conditions (such as ataxia, neuropathy, depression, etc.) [1]. The diagnosis is based on the integration of data coming from serologic tests, such as tissue transglutaminase (IgA-TG2), deamidated gliadin peptides (IgG-DGP) and endomysium (IgA-EMA) antibodies, endoscopic findings, and HLA-DQ2/8 typing to rule out genetic vulnerability [1]. The gold standard for the diagnosis of Celiac disease is the duodenal biopsy, or in the pediatric age, the simultaneous detection at the same time of anti-transglutaminase antibodies > 10 normal values and positive anti-endomysial antibodies according to the latest ESPGHAN guidelines [1,18]. The only validated therapy for CD is adherence to a gluten free diet (GFD); in almost all celiac patients who follow a GFD, the symptoms disappear, and the intestinal damages progressively improve [2,17]. According to the European Commission, a food to be labelled as gluten-free must contain a maximum of 20 ppm of gluten [1]. It has been reported that non-adherence to a gluten-free diet increases the risk of malignancies and cardio-vascular diseases, decreased bone mineral density, and worse pregnancy outcomes [1]. While CD is a well-known health condition, much debate remains around whether gluten proteins can trigger symptoms in patients with no features of CD, so called non-Celiac gluten sensitivity (NCGS), which can be suspected when both CD and wheat allergy are ruled out [1,3,4,19]. These patients suffering from gastrointestinal discomfort and extra-intestinal manifestations do not present CD serology and histopathology, but they complain of the same symptoms of Celiac patients, which improve after following a gluten-free diet [1,6,8]. Moreover, in contrast to CD, patients with NCGS rarely manifest malabsorption, and they do not have an increased risk of malignancies [1].

NCGS prevalence is unknown, and its etiopathogenesis is still unclear, and it may be the result of an innate and adaptive immune response activation consequent to gluten exposure in patients with altered intestinal barrier [1,19]. However, many studies report that amylase, trypsin inhibitors (ATIs), and FODMAPs contained in wheat can also trigger the symptoms, just as with gluten, thus making this syndrome vague [19]. Furthermore, unlike CD and wheat allergy, up to this day there are no serological or histopathological findings that can confirm the diagnosis [17]. In order to verify the hypothesis that gluten may cause this condition, several authors conducted randomized placebo-controlled trials in adult and pediatric populations [20,21]. The therapy is represented by a GFD [22]. However, NCGS is not associated with CD characteristic complications, so the GFD does not have to be as strict as the one of celiac patients, since the aim of the diet is to obtain symptomatic relief [22]. The last gluten-related disorder is wheat allergy, which affects 0.5–9% of the general population, even though it is more common in pre-school children [2,17]. In these patients, the ingestion, contact, or inhalation of wheat and wheat products leads to different and various clinical presentations, such as occupational asthma and rhinitis (baker’s asthma), food allergy (FA), contact urticaria, and wheat-dependent exercise-induced anaphylaxis, which is an anaphylactic reaction after the ingestion of a product containing wheat followed by physical exercise [8,17]. A T helper cell type 2 activation is involved in wheat allergy pathogenesis, thus stimulating the production of IgE antibodies by B cells [3,4]. Once the wheat allergens are recognised and bonded to the specific IgE antibodies, their cross-linking leads to a hypersensitive reaction and to the activation of mast cells and basophils [6]. The symptoms develop within minutes to hours after the ingestion of the insoluble gliadins contained in wheat [3,4]. The clinical presentation varies and can involve different organs and systems, such as the cardiocirculatory, the respiratory, the gastro-intestinal ones, and the skin, leading to the possible appearance of rash, itching, and angioedema, up to life threatening events, such as anaphylaxis [3,4,21]. The diagnosis relies on clinical history, skin prick test, specific IgE-antibodies levels and, if necessary, functional tests, such as the oral food challenge [17].

The therapy of wheat allergy primarily consists of wheat and wheat products avoidance; generally, it is not necessary to exclude rye and barley from the diet, even if events of cross-reactivity are possible [6]. Nevertheless, it is important to underline that, unlike celiac patients, many children affected by immediate wheat allergy recover by school-age, while wheat allergy is usually permanent in adults [17,23].

Table 1 summarizes the main characteristics of gluten-related diseases.

## 5. The Impact of Gluten-Free Diet on Gut Microbiota in Gluten Related Diseases

The importance of gut microbiota in celiac disease, non-celiac gluten sensitivity, and wheat allergy is acknowledged, even if its exact role in these diseases’ aetiopathogenesis is still unclear.

Before the diagnosis, Celiac patients present an altered intestinal microflora, characterized by an increased number of total bacteria and a raised percentage of harmful ones [24]. However, it is not fully understood if these alterations are an effect of the disease itself or might contribute to its development, leading to an increased intestinal permeability, an altered gluten processing, or an immunomodulatory effect [24,25,26]; it certainly concurs on inappropriate functioning of bowels and is related to the severity of clinical symptoms [27]. Microbiota play an important role in the stimulation and regulation of the immune system [28]. Different mechanisms of microbiota immunomodulation have been described in several studies. Short chain fatty acids (SCFA) that are produced by microbiota’s components affect Treg cells [29]. The abnormal butyrate production by microbiome is recognised as a cause of higher expression of non-functional form of FOXP3, which is associated with an enlarged risk of autoimmunity [30]. Many studies agree that the microbiota in patients treated with a GFD is more similar to healthy gut microbiome in comparison to patients consuming gluten [31,32,33]. These findings support the occurrence of gut dysbiosis in CD, which improves following gluten withdrawal. In celiac patients, who do not follow a GFD, microbiota is abundant with pro-inflammatory Gram-negative species, such as Proteobacteria, Bacterioides, Prevotella, ecc., while it is poor in probiotic ones, such as Firmicutes, Actinobacteria, Bifidobacteria, Lactobacilli, and Streptococceae, which have instead a protective role [24,34,35,36,37,38,39,40].

In order to understand the exact role of these bacterial species in CD pathogenesis, Caminero et al. colonized germ-free mice with bacteria found in Celiac patients’ and healthy individuals’ guts [41]. They saw that *Pseudomonas* spp., which is abundant in CD patients, was responsible for an alteration of the intestinal mucosal barrier, as well as an activation of specific T-cells, while *Lactobacillus* spp., which was prevalent in healthy controls’ microbiota, splitted gluten peptides, thus reducing their immunogenicity [41]. The protective role of *Lactobacillus* spp. was confirmed even by the work of Herrán et al., which underlined how this bacterium is involved in the gluten digestion process [42].

To the contrary, several studies showed that a good adherence to a gluten-free diet in CD patients can partly restore the normal composition of the microbiota [24,26].

While in the literature there are many studies regarding the correlation between gut microbiota and CD, fewer studies were drafted about gut microflora and NCGS and its possible role in the disease pathogenesis [24,43,44]. At baseline, Garcia-Mazcorro et al. found an increased number of Actinobacillus and Ruminococcaceae genera in NCGS patients compared to Celiac ones and a higher rates of Pseudomonas spp. after gluten withdrawal from the diet [45]. This suggests a possible protective role of some members of the Pseudomonas family; however, there are not so many studies about the exact role of native-gut associated Pseudomonas [45]. The work of Nobel et al., confirmed, at baseline, gut microbiota alterations in CD and NCGS patients compared to healthy controls. However, a 14-day gluten trial did not lead to any modifications of the gut microflora in these patients, who already had an altered microbiota diversity [46]. This might suggest that periodic and brief gluten exposures do not interfere with the gut microbiome [46]. The data collected so far agree that both CD and NCGS patients present various degrees of dysbiosis, which can contribute to the pathogenesis of these two gluten-related diseases [24]. Almost all the studies examined [45,47,48] agree on the beneficial role of GFD on symptoms improvement, which is the cornerstone therapy of these disorders; however, its impact on gut dysbiosis is controversial, since it can only partially restore the normal gut microbiota and, on the other hand, it decreases bacterial biodiversity [24]. While the studies regarding gut microbiota in Celiac patients are copious and many are being written about its changes in NCGS patients, the literature we reviewed is still poor in works about the association between gut microflora and specific food allergies, such as wheat allergy [49,50]. Further studies are necessary in order to better evaluate the correlation between gluten-free diet and gut microbiota.

## 6. Gluten and Inflammatory Bowel Diseases

Inflammatory bowel diseases (IBD) are chronic inflammatory relapsing and remitting conditions that affect the gastrointestinal (GI) tract [7,51]. Current knowledge recognizes diet both as a risk factor for IBD development due to its role in inducing intestinal dysbiosis and aberrant mucosal immune response in genetically predisposed individuals, but also, as a potential tool in the management of these diseases [52,53]. As we discussed above, gluten might be one of the main players in this interplay due to its role in intestinal inflammation. Indeed, Celiac disease, which is a chronic immune-mediated enteropathy consequent to gluten intake, shares with IBD a multifactorial etiology, resulting from a complex interplay between genetic variability, environmental factors, and dysregulated immune response [44].

These pathogenic and epidemiological similarities are widely confirmed in the literature we reviewed, as seen in the work of Aghamomadi et al., who observed a similar expression pattern of pro-inflammatory cytokines in intestinal biopsy both in CD and IBD patients, proposing new prospectives to find common therapeutic targets for these diseases, but also novel common potential biomarkers for their diagnosis [54]. Shah et al. also pointed to CD as a potential risk factor for IBD; indeed, this systematic review demonstrated an increased risk of developing IBD in patients with CD as compared to the general population [55]. The precise mechanism of this association is yet to be explored, but it appears likely that the CD-associated mucosal inflammation initiates a sequel of events that ultimately result in IBD [55]. Recently, Bramuzzo et al. showed that children with both IBD and CD have peculiar phenotypic features with a higher risk for autoimmune diseases, colectomy, and pubertal delay compared with IBD alone [56]. Additionally, the study conducted by Yehuda et al. showed that IBD patients had a higher prevalence of other autoimmune diseases, including CD, compared to non-IBD patients [57]. Accordingly, Alkhayyat et al., in their retrospective analysis, highlighted a significant risk association between CD and IBD (OR, 13.680; 95% CI, 13.454–13.909, *p* < 0.0001); moreover, the risk of developing CD was higher compared to the risk of occurrence of other autoimmune diseases, in particular in ulcerative colitis [58]. To the contrary, in the study conducted by Casella et al. on 1711 IBD adults, only 0.5% of them had a diagnosis of CD confirmed both histologically and serologically, thus making this connection poor and confirming the need of further investigations in order to make more precise statements [59].

Given the role of gluten in intestinal inflammation, we also tried to investigate the impact of gluten intake on IBD symptom’s onset or exacerbation. In the literature, we reviewed, in fact, that it is reported that more than half of patients with IBD believe that their symptoms are induced or exacerbated by specific foods, in particular gluten [51]. At the moment, a GFD is not recommended in IBD patients. However, different studies have discussed its use in alleviating GI symptoms, even if there is still no scientific evidence of the beneficial role of eliminating gluten from the diet [5]. The study by Morton et al. on 233 IBD patients, who completed a self-administered retrospective questionnaire, reported that 66% of the patients presented an improvement of the symptoms, and 38% presented a reduced flare frequency and severity after following a GFD trial [51]. This finding was also observed in the study by Triggs et al. focused on food intolerances [60]. The majority of the subjects (>66%) in the study population reported that specific changes in their diet permitted a decrease in either the number of flares or the severity of gastrointestinal symptoms [60]. Gluten-free products, in particular, were reported to be frequently associated with a reduction of the symptoms and were least associated with adverse effects [60]. Accordingly, Herfarth et al. performed a cross-sectional study in which they administered a dietary questionnaire regarding GFD adherence to 1647 patients affected by IBD [61]. They observed that a substantial number of the study population previously tried (19.1%), or was currently following, a GFD (8.2%, 135 patients) [61]. Adherence to the GFD was associated with fatigue reduction and an important improvement of at least one GI symptom (e.g., bloating, diarrhea, abdominal pain, nausea) in 65.5% of these patients [2,61]. Moreover, 38.3% of the patients reported a decrease in disease flare frequency and severity, and 23.6% of them needed less IBD medications [61]. On the other hand, Zallot et al., in their study, proposed a 14-item questionnaire to 244 IBD adults, and even if 9.5% of these patients believed that a GFD was helpful in improving their symptoms during disease’s flares, only 1.6% actually decided to adopt a GFD during a disease relapse [62]. Accordingly, Schreiner et al. conducted a study on 1254 IBD patients and concluded that the patients who were following a GFD (4.7%) did not report differences in disease activity, complications, and hospitalization rate compared to patients who did not follow a specific dietary regimen [63]. They also highlighted a worse psychological wellbeing in those who were following a GFD diet [63]. These recent contradictory findings highlight the importance of further studies in order to evaluate the possibility of proposing a GFD in patients affected by IBD in clinical practice.

Table 2 summarizes the main studies regarding the role of gluten and the impact of GFD on IBD.

## 7. Gluten and Functional Gastrointestinal Diseases

Functional gastrointestinal diseases (FGIDs) are characterized by chronic or recurrent GI symptoms not sustained by any organic or biochemicals disorders [64]. They affect up to 40% of the population globally, and their prevalence is approximately 23% in the pediatric population of the Mediterranean area [64,65,66,67]. The pathogenesis of FGIDs is multifactorial and is still to be defined yet [64]. Genetic factors, gastrointestinal motility disturbances, brain–gut axis alterations, psychological factors, and visceral hyperalgesia are just some of the several mechanisms which may contribute to their pathogenesis [64]. Currently, both pediatric and adult FGIDs are described within the Rome IV criteria [64,65]. The adherence to specific dietary patterns and the assumption of certain foods, particularly gluten, has been frequently associated with the development and persistence of FGIDs, even if this link is yet to be properly defined [65]. Even without a proper CD diagnosis, some patients affected by FGIDs avoid gluten intake, since it has been reported to trigger or worsen some GI symptoms [65].

Functional symptoms and, in particular, functional dyspepsia are, in fact, frequently reported by Celiac patients; therefore, we firstly described paper evaluating the risk of developing CD in patients with functional dyspepsia (FD) and vice versa, as well as the prevalence of FD in CD patients [68,69]. As described by Lasa et al. in their case-control study of 320 patients with FD, CD was diagnosed in 1.25% of the dyspeptic patients versus 0.62% of the controls [69] concluding that there was not an increased risk of CD for patients with functional dyspepsia when compared to healthy individuals [69]. Santolaria et al. instead suggested that CD could be more prevalent in patients with FD [70]. In their study in which they gathered 142 patients affected by functional dyspepsia, they observed that 35.9% of them had an enteropathy confirmed by duodenal biopsy and a CD diagnosis was stated in 19.7% of these patients [70]. On the other hand, the prevalence of FGIDs, including FD in Celiac patients, was described in the works of Cristofrori et al. and Silvester et al. [71,72]. Cristofrori et al. instead conducted a prospective cohort study on 417 children affected by CD who had been on GFD for more than a year and had positive serological response to the diet [71]. They observed that the prevalence of functional abdominal pain (FAP) was higher in CD patients (11.5%) compared to the controls, but also irritable bowel syndrome (7.2%) and functional constipation (19.9%) were more prevalent in Celiac patients compared to the control group (3.2% IBS, 10.5% FC) [71]. The study of Silvester et al. gathered a group of 119 adult CD patients newly diagnosed and observed that 27% and 52% of CD subjects fulfilled the criteria for FD and IBS diagnosis, respectively [72]. The prevalence of those meeting IBS and FD symptom criteria both decreased significantly after a year of GFD, to 22% and 8%, respectively [72]. However, they found a high prevalence of functional symptoms even after a year of GFD (47%) [72]. This significative reduction of functional disorders prevalence after gluten withdrawal from the diet might lead the reader to ask if the functional symptoms were a display of the CD itself. Indeed, the patients were on a GFD only for one year, and there was not a control group.

There are several works that analyze the effect of GFD on GI symptoms in FGIDs. We first described the effect of GFD on functional dyspepsia, which is one of the main FGIDs associated with gluten intake [73], as described above. Du et al., in their prospective-observational study, enrolled 101 newly diagnosed FD patients and 31 asymptomatic controls and evaluated their dietary gluten consumption through self-reported questionnaires [73]. They observed that, among patients with FD, the assumption of foods rich in gluten led to the symptoms’ onset and worsened them, in particular early satiety [73]. Accordingly, Elli et al. enrolled 140 FGIDs patients, 22 of which were diagnosed with FD that received a strict three-week GFD [74]. The patients who positively responded to the GFD were submitted to a placebo-controlled double-blind gluten challenge with crossover [74]. Up to 75% of the FD patients positively responded after the first phase, showing an improvement of general wellbeing [74]. However, after the placebo-controlled gluten rechallenge, 14% of these responder patients had an exacerbation of the symptoms, leading the authors to hypothesize a potential placebo effect of GFD, but also wondering about the role of another foods in symptoms’ exacerbation, in particular wheat components, such as FODMAPs [74]. In addition, Shahbazhani et al. investigated the effect of a six-week GFD trial on 77 patients with refractory FD and observed that just one in three patients positively responded to the GFD in terms of clinical improvement, while 6.4% of the patients could be labelled as affected by NCGS, as they manifested symptoms after blind gluten ingestion [75].

Regarding the effect of GFD on the other FGIDs, Aziz et al. evaluated the change in GI symptoms in 41 adults with IBS-diarrhoea who were asked to follow a GFD for six weeks; the study also included an evaluation of the patients’ HLA-DQ2/8 status [76]. They observed that 71% of the patients showed a significant improvement of the IBS-Symptom Severity Score (IBS-SSS), irrespective of the HLA-DQ2/8 haplotype [76]. Accordingly, Barmeyer et al. proposed a four-month GFD to 35 IBS patients and observed that 34% of the subjects were responders to the diet, as they had significant symptoms and wellbeing improvement, thus highlighting no association with HLA-DQ2/8 expression [77]. Zanwar et al., in a double-blind randomized placebo-controlled trial (DBCPT), enrolled 60 adults with IBS in order to evaluate their clinical response to gluten administration through the use of VAS scale score [78]. In the first phase, all subjects had to follow a GFD for four weeks, and those who had a positive response were included in a double-blind gluten rechallenge after a four-week washout [78]. The group who received gluten reported an important worsening of all gastro-intestinal symptoms, with the exception of flatulence, compared to the placebo group [78]. Additionally, Biesiekiersky et al. conducted a double-blind randomized placebo-controlled trial (DBPCT), involving 34 patients with IBS in which CD was ruled out [21]. They observed that patients who received gluten had higher severity scores over the entire study period and much greater changes in overall symptoms compared to the placebo group; this evidence suggested that IBS patients could benefit from a GFD even without a gluten intolerance diagnosis [21]. Moreover, Wahnschaffe et al. conducted a prospective trial on 41 adults with IBS who followed a GFD for six months opposite to 102 healthy volunteers as control group [79]. After following a GFD, 41 IBS patients showed a significant decrease in GI symptom and stool frequency and score, which, in 49% of the patients, improved within the normal range [79]. A normal GI symptom score achievement, after following a GFD, was more frequently observed in HLA-DQ2 positive patients and/or in patients who had Celiac disease-associated IgG antibodies [79]. Accordingly, in a single-centre randomized interventional trial conducted by Vazquez-Roche et al. on 45 IBS patients who followed either a gluten-contained diet (GCD) or a GFD for four weeks, it was observed that a significant increase in stool frequency and small bowel permeability in the GCD group occurred, in particular in HLA-DQ2/8 positive subjects [80]. Nevertheless, no beneficial diet effect was observed on gastric emptying, colonic transit, and stool form [80].

These studies show that there is still controversy about this subject, and further controlled dietary interventional studies on the use of GFD in FGIDs patients are required. In the literature we reviewed, in fact, some of the FGIDs patients showed an improvement in their symptoms and wellbeing after some weeks of strict adherence to GFD, but on the other hand, others showed a relapse of their symptoms after the double-blind re-challenge with gluten, making it possible to hypnotize a NCGS diagnosis in these patients on top of their previous FGIDs diagnosis.

Table 3 shows the main studies regarding the role of gluten and the impact of GFD on FGIDs.

## 8. Conclusions

The progressive increase in gluten-intake led several authors to investigate its role in triggering and perpetuating some gastrointestinal disorders. As mentioned above, there are three main gluten-related diseases: CD, non-celiac gluten sensitivity, and wheat allergy. Even if the common thread is represented by gluten, these health conditions differ in etiopathogenesis, diagnosis, and therapy. Following a strict life-long gluten free diet is the cornerstone therapy for celiac patients, who otherwise have an increased risk of developing complications.

Unlike celiac patients, people who suffer from NCGS should follow a gluten free diet in order to improve their symptoms, but it does not have to be life-long and as strict as the one of CD patients, since the aim of this dietary scheme is to obtain symptomatic relief. Another interesting point comes from the many studies which report that amylase, trypsin inhibitors (ATIs), and FODMAPs contained in wheat can trigger the symptoms, just as with gluten, thus opening the way to new therapeutic options. Differently from CD and NCGS, wheat allergy is a stand-alone entity, since the clinical manifestations appear after the ingestion, inhalation, or contact of wheat and are not associated to gluten contained in rye or barley, unless events of cross-reactivity happen.

At present, no specific nutritional regimen has been proven to be completely effective both in IBD and in FGIDs, even though the role of many nutrients, in particular wheat and gluten, in shaping the intestinal microbiome and influencing intestinal inflammation has been widely studied. Nowadays, there is still insufficient evidence to recommend gluten’s restriction in these patients’ diet, mostly because gluten is most likely only one of the many factors involved in the occurrence of clinical symptoms. On the positive side, following a GFD in clinical practice has the potential to be an easy and efficient therapeutic approach when it comes to verify its effect on clinical outcomes and to identify NCGS patients not previously diagnosed. Moreover, especially during the disease’s flares, most patients intentionally avoid some nutrients from their diet, which they point to as the cause of their symptoms. Some patients, in fact, tend to avoid gluten spontaneously, despite not having a CD diagnosis, even though these dietary restrictions can be very severe. However, gluten-free diet is an unbalanced dietary pattern and the strict adherence to it can lead to micronutrients (minerals, vitamins) and dietary fibre deficiencies, unless the dietary scheme is properly supervised by a nutritionist. Gluten-free manufactured foods are rich in salt, fats, and sugar in order to make them more palatable for the costumers; this composition has poor quality nutritional properties, and it has been linked to a possible increase in cardiovascular risk and also can predispose to inflammatory and functional gastrointestinal diseases through the modifications of the gut microbiota.

Finally, GFD can have a negative impact on the psychological wellbeing of these patients secondary to the restrictive characteristics of the diet itself. The understanding of an individualized diet that could modulate the composition and metabolism of gut microbiota, achieve the disease’s remission, and, possibly, maintain an optimal homeostasis and prevent any flare, which is the actual therapeutic goal. Even though these data are intriguing, further studies are needed before gluten avoidance can be recommended.

## Figures and Tables

**Table 1:** Main characteristics of the three different gluten-related diseases: Celiac disease (CD), non-Celiac gluten sensitivity (NCGS), and wheat allergy (WA).

	Celiac Disease (CD)	Non Celiac Gluten Sensitivity (NCGS)	Wheat Allergy (WA)
**Prevalence**	1% of the general population	Unknown	0.5–9% of the general population (>in children)
**HLA-DQ2/8 haplotypes**	Positive	Positive in 50% of the patients	Negative
**Pathogenesis**	Autoimmune	Activation of the innate and adaptive immune response	IgE-mediated
**Serological markers** **Duodenal biopsy**	Positive IgA-TG2, IgA-EMA and IgA/IgG-DGP antibodies Necessary *	IgA/IgG anti-gliadin positive in 50% of the patients Necessary to rule out CD	Wheat specific IgE antibodies or prick test Not necessary
**Therapy**	Strict gluten free diet	Gluten-free diet	Avoid all contact with wheat

* In the latest ESPGHAN guidelines, duodenal biopsy is not necessary in pediatric patients who have positive anti-TG2 antibodies (>10 times) and positive anti-EMA antibodies in a second sample. (ESPGHAN) [18].

**Table 2:** Main characteristics of the studies regarding gluten intake or use of a gluten free diet in inflammatory bowel diseases.

Study	Type of Study	Population	Aim	Results
Triggs et al. [60], 2010	Observational study	An amount of 446 subjects with Crohn’s disease	Evaluation of the impact of GFD in IBD patients	Decreased number of flares and GI symptoms’ severity following a GFD (66%)
Zallot et al. [62], 2013	Observational study	An amount of 244 IBD patients	Evaluation of the impact of diet in IBD patients	An amount of 57.8% of the patients felt that food could play a pivotal role in IBD flares; 9.5% of patients believed that a GFD was helpful in improving their symptoms during the disease’s flares; only 1.6% of the study population decided to adopt a GFD during disease flares;
Herfarth et al. [61], 2014	Cross-sectional study	An amount of 1647 IBD patients	Investigate the adherence to GFD among IBD patients and their experience with it	An amount of 19.1% of the study population tried a GFD, while 8.2% of them were already following it. After following a GFD, 65.6% of the patients reported an improvement of the symptoms, 38.3% a reduction of disease flares’ frequency and severity, and 23.6% needed less medications
Schreiner et al. [63], 2019	Prospective study	An amount of 1254 IBD patients	Investigate the adherence to GFD among IBD patients and their experience with it	An amount of 4.7% of the study population followed a GFD and did not find any differences in disease activity, complications, and hospitalization rate Worse psychological wellbeing in those who were following a GFD diet
Morton et al. [51], 2020	Observational study	An amount of 233 IBD patients	Evaluation of the effects of GFD trial on IBD symptoms and flares	An amount of 66% of the patients reported an improvement of the symptoms and 38% reported reduced flare frequency and severity

**Table 3:** Main characteristics of the studies regarding gluten intake or use of a gluten free diet in FGIDs.

Study	Type of Study	Population	Aim	Results
Wahnschaffe et al. [79], 2007	Prospective interventional study	An amount of 145 IBS adult patients. 102 healthy controls	To evaluate the symptoms’ response to a GFD in IBS patients.	An amount of 41 IBS patients showed a significant decrease in stool frequency and GI symptom score, which in 49% of the patients improved within the normal range.
Biesiekierski et al. [21], 2011	Double-blind randomized placebo-controlled trial	An amount of 34 IBS adult patients, who ruled out CD.	Determine whether gluten ingestion could induce symptoms in non-celiac individuals affected by IBS	The majority (68%) of the patients who received gluten had higher severity scores over the entire study period and much greater changes in overall symptoms compared to the placebo group.
Vazquez-Roque et al. [80], 2013	Single-centre randomized controlled trial.	An amount of 45 IBS adult patients.	Evaluation of the impact of a four-week GFD randomly administered in IBS patients according to HLA haplotype.	Significant increase in stool frequency and small bowel permeability in the GCD group, especially in HLA-DQ2/8 positive subjects. No beneficial diet effect was observed on gastric emptying, colonic transit, and stool form.
Aziz et al. [76], 2016	Prospective interventional study.	An amount of 41 patients with IBS-D (20 HLA-DQ2/8 positive and 21 HLA-DQ2/8 negative).	Evaluation of the effects of a six-week GFD in patients with IBS-D, according toHLA haplotype.	Reduction of IBS Symptom Severity Score by 50 points in 29 patients (71%). The mean total IBS Symptom Severity Score decreased from 286 to 131 points after six weeks of diet (*p* < 0.001); the reduction was similar in each HLA-DQ group.
Zanwar et al. [78], 2016	Double-blind randomized placebo-controlled trial.	An amount of 60 IBS adult patients who ruled out CD and wheat allergy.	Evaluation of the effects of GFD in IBS patients. Subsequent gluten rechallenges after four weeks of washout in patients who positively responded.	In the first phase, overall symptom VAS score improved for 36% of patients after GFD. After four weeks of food rechallenge, patients in the gluten group presented worsening of symptoms, with higher weekly median overall symptom VAS, in contrast to those in the placebo group (*p* < 0.05) Patients who followed a gluten contained diet showed higher rates in terms of bloating, abdominal pain, and tiredness.
Elli et al. [74], 2016	Multi-centre randomized double-blind placebo-controlled study.	An amount of 140 FGIDs patients	Evaluation of the impact of GFD on FGIDs. Patients who positively responded to a strict three-week GFD underwent a placebo controlled double-blind gluten challenge with crossover	Up to 75% of the patients positively responded to the three-week GFD, showing an improvement of general wellbeing. After the placebo-controlled gluten challenge, 14% of these responder patients had an exacerbation of the symptoms, suggesting a NCGS diagnosis and a possible placebo effect of GFD
Barmeyer et al. [77], 2016	Prospective interventional study.	An amount of 35 IBS patients	Evaluation of the impact of a four-month GFD on IBS symptoms according to HLA haplotype.	An amount of 12 patients (34%) had significant symptoms and wellbeing improvement, showing no association with HLA-DQ2/8 expression.
Du et al. [73], 2017	Case-control study.	An amount of 101 newly diagnosed FD patients and 31 asymptomatic controls	Evaluation of the impact of gluten assumption on FD	The assumption of foods rich in gluten was higher in frequency (*p* = 0.047) and quantity (*p* = 0.01) in FD patients compared to controls. Early satiety (*p* = 0.03) and worsening of GI symptoms were associated with increased gluten consumption.
Shahbazkhani et al. [75], 2020	Randomized double-blind placebo controlled trial.	An amount of 77 patients with refractory FD.	Evaluating the effect of GFD in refractory dyspeptic patients. Patients who responded to the GFD underwent a double-blind placebo-controlled gluten challenge.	An amount of 35% of the patients positively responded to the GFD in terms of clinical improvement. An amount of 6.4% of the patients could be labelled as affected by NCGS, as they manifested symptoms after blind gluten ingestion.

## Data Availability

Not applicable.

## Abbreviations

Celiac Disease: CD; Non-Celiac Gluten Sensitivity: NCGS; Wheat Allergy: WA; Food Allergy: FA; Gluten Free Diet: GFD; Inflammatory Bowel Diseases: IBD; Ulcerative Colitis: UC; Crohn’s Disease: CD; Fermentable, Oligo-, Di-, Mono-saccharides And Polyols: FODMAP; Functional Gastrointestinal Diseases: FGIDs; Irritable Bowel Syndrome: IBS; Functional Dyspepsia: FD; Functional Constipation: FC; Tumour Necrosis Factor-α: TNF-α, Gastro-Intestinal: GI, Double-Blind Placebo-Controlled Trial: DBPCT.
